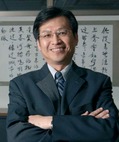# Review series – Mechanotransduction from physiology to disease states

**DOI:** 10.1111/jcmm.12037

**Published:** 2013-02-27

**Authors:** Chao-Min Cheng, Ming-Jer Tang

**Affiliations:** Institute of Nanoengineering and Microsystems National Tsing Hua UniversityHsinchu 300, Taiwan; Department of Life Science, Tunghai UniversityTaichung 407, Taiwan

Traditional boundaries between engineering and life sciences are rapidly disintegrating as interdisciplinary research teams develop new engineering tools for exploring fundamental issues in both medicine and biology. The initial innovation for biomechanics was primarily developed to study the function of human tissues. With recent technological advances in multiple research fields such as cell biology, molecular biology, as well as micro-/nanotechnology, much attention is shifting to probe the functionality of mechanics at the cellular and molecular levels. The pursuit of this direction helps to reveal the mechanisms of and therapeutic potentials for some of the most lethal diseases, including cardiovascular diseases, organ fibrosis and cancer. This interdisciplinary approach has generated great interests among researchers working in a wide variety of communities, including industry, universities and research laboratories. In this connection, a fair amount of evidence has been recently accumulated to indicate that physical-based stimuli (*e.g*. the mechanical interactions between cells and their microenvironments) play a crucial role in cancer biology with the developments of various physical, chemical or biological approaches.

We initialized discussions with Prof. Laurentiu M. Popescu, the Editor-in-Chief of *Journal of Cellular and Molecular Medicine*, about the organization of a ‘Review Series’ in 2012, focusing on several specific issues in cellular and molecular biomechanics (mechanobiology). The issues regarding mechanobiology in this Review Series that we have addressed cover physiology (cardiac physiology, haemostasis and thrombosis and neurosensory mechanotransduction), cell biology (cell adhesion and migration, stem cell differentiation) and pathology (cancer development, skin disorders and degeneration and vascular pathobiology). Here, we would like to show our deep appreciations to all authors and reviewers. Without their greatest help and contributions, this Review Series would not be published on time. This Review Series may not cover all issues in this emerging scientific field. However, we believe that our efforts have a great potential ‘to hurl a boulder to draw a jade (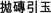)' and ignite the innovations and challenging discussions in this field.

Chao-Min Cheng received his Ph.D. in 2009 from Carnegie Mellon University (Biomedical Engineering Department); he then did his post-doctoral training with Prof. George M. Whitesides at Harvard University to develop paper diagnostic systems for global public health. He is currently an independent P.I. at National Tsing Hua University, Taiwan, starting from 2011 summer. He has been blessed to receive the Traveling Fellowship in Journal of Cell Science, and Distinguished Young Investigator Research Grant from National Science Council in Taiwan. He was also an invited attendee for NAS Sackler Colloquium at the National Academy of Sciences, and research highlighted in the National Academies – Keck Futures Initiative, Scientific American, Chemistry World and New York Times. His general research interests focus on cellular and molecular biomechanics and mechanotransduction, paper diagnostic systems for public health (including the development of sensing elements) and biomedical devices for cellular and molecular biology. He is also currently an acting member for International Affairs/Globalization Committee at Biomedical Engineering Society (BMES) and Editorial Board member in *Sensor Letters*.


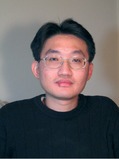


Ming-Jer Tang is currently the Distinguished Professor of Physiology, National Cheng Kung University Medical College, Tainan, Taiwan. He received his M.D. from Taipei Medical College, Taiwan in 1980 and then Ph.D. in Physiology from University of Michigan, Ann Arbor in 1987. After 3 years Post-doctoral Fellowship at UM and University of Southern California Medical School, he came back to his hometown and joined National Cheng Kung University in 1990. He was the Associate Professor from 1990 to 1996, Professor from 1996 to 2002 and Distinguished Professor since 2002 in Department of Physiology, National Cheng Kung University Medical College. His research and teaching interests have been renal physiology, tissue engineering and regenerative medicine. His laboratory has unravelled the functions of and interactions between two collagen receptors, *i.e*. α2β1 integrin and DDR1, in epithelial cell differentiation. His current research is to explore mechanobiology of cancer and fibrosis of the tissues. He has a long-term experience in academic administration. He was Executive Vice Dean of National Cheng Kung University Medical College from 2001 to 2007 and Vice President of Academic Affairs for National Cheng Kung University from 2007 to 2011. He is now President of Tunghai University, the first Liberal Arts University in Taiwan.